# Supplementary material for: Occurrence, diversity and distribution of Trypanosoma infections in cattle around the Akagera National Park, Rwanda
Source: PLoS Negl Trop Dis. 2021 Dec 15;15(12):e0009929. doi: 10.1371/journal.pntd.0009929 (PMC8726506; doi:10.1371/journal.pntd.0009929)
Supplement: Attachment — Submitted filename: PNTD-D-21-00831_COMMENTS.docx [file pntd.0009929.s002.docx]

Occurrence, diversity, and distribution of Trypanosoma infections in cattle around the Akagera National Park, Rwanda by Gashururu *et al.*

**Reviewer’s summary**

Gashururu *et al* set out to determine the prevalence and genetic diversity of different trypanosome species circulating in cattle from the wildlife-livestock interface areas of the Akagera National Park, Rwanda. They used an array of parasitological, serological, and molecular techniques to screen 1,037 cattle blood samples for different Trypanosome species: hence detecting *T. congolense, T. vivax*, *T brucei brucei*, and *T. evansi* as the economically important Trypanosome species infecting cattle around the Akagera National Park, Rwanda. They further reported these trypanosome isolates to be 98.7-100 % identical to those previously isolated from the East African region. They discuss these data sets with respect to the epidemiology of AAT in the area and the potential of these data sets in informing control programs aimed at progressively reducing and ultimately eradicating AAT from Rwanda.

This manuscript presents somewhat large survey of AAT in the region using different diagnostic techniques; datasets that I deem helpful in broadening the understanding of the epidemiology of AAT in Rwanda. These data sets are also deemed necessary in designing and implementation AAT control programs in the area. This is a good manuscript in its area which would further be improved by amending it following the suggested the major and minor changes below; -

A. **Major changes [non-discretionary]**
1 **Design/methods**
i. Gashururu *et al* need to include a section of sample size determination. I hope the readers should be to know some of the key parameters that were considered to calculate the sample size n=1,037. These parameters might include but are not limited to;-
a) Number of cattle in the area
b) sectors within districts and number of cattle containing sectors in the study area
c) total number of farming households within the study area
d) Type of sampling [e.g., simple random sampling or cluster sampling]
e) If cluster sampling was adopted, what was the total number of clusters in the study area and what is the definition of the cluster? What was the minimum number of clusters that was picked to satisfy the set precision? How many animals were picked from each cluster?
f) Average number of cattle per cluster
g) sampling units/clusters [e.g., herds, or district sectors or even individual animals] and sampling fraction
h) if cluster sampling was adopted what was the intercluster correlation coefficient used?
i) background prevalence of AAT in the area if known or was a 50% baseline prevalence assumed?
j) precision of the sample estimate (one half-length of the 95% confidence interval)
k) design effect
l) selection of each sampled animal; making sure that each animal was given an equal opportunity to be included in the survey, if indeed animals were selected randomly.
m) other [predictors of infection] data that was collected accompanying the blood samples e.g., age, sex, breed, predisposing factors [e.g., use of pyrethroid insecticides and trypanocides]
n) numbers and categories of other livestock that could be reservoirs of human infective trypanosomes or whose production/health might be compromised by AAT
o) define what indigenous, exotic, and cross breeds are and the sampling fraction for each of these breeds [i.e., total in the study area vs those that were sampled]. A good description of the inclusion criterion. Refer to lines 157-158; Please provide justification /reference for this.
i. Motivate the readers in the background and methods sections why it was necessary to use microscopy [Thin & Buffy coat smears] and VerYDiag to detect trypanosomes or current/previous exposure to Trypanosomes. What is the value of comparing HRM-PCR, Microscopy [Thin & Buffy coat smears] and VerYDiag? Is it not obvious that HRM-PCR is better than microscopy and VerYDiag in detecting different trypanosomes? The deployment of the selected panel of diagnostic techniques is not discussed either.
ii. Given the multiplicity of the diagnostic/ analytical procedures undertaken, it would be beneficial to include a schematic study flow diagram so that readers can quickly visualise what was done to arrive at the results presented herein.
iii. The authors need to consider undertaking phylogenetic analysis [Gamma distribution with 1000 bootstrap replicates] of the nucleic acid sequences of Trypanosome species in this study area and those from the region [East Africa or Africa]. In addition, it would be nice to evaluate sequence divergence [pairwise genetic distances] of Trypanosome species in this study and their corresponding reference sequences
iv. I would like to suggest that Figure 3 is revisited with a view to map the spatial distribution of *T. vivax,* *T. congolense*, *Trypanozoon* species and *T. theileri* using either of the following strategies
a) Aggregated farm level prevalence of different Trypanosome species detected by the most sensitive /specific diagnostic test or an average of all tests; with the diameter of the circle showing prevalence proportional to sampled animals or prevalence symbology stratified like 0-5, 5-10, 10-15, 15-20; with increasing size of the symbology. If the number of sampled farms is too many, you can aggregate these data sets at sector level
b) You can map presence and absence of different species, where presence and absence are as determined by all diagnostic tests or the most sensitive/specific test

Note that option (a) is more intuitive than option (b)

2. **Results**
i. Please include results related to comment 1 (iv) above, i.e., phylogenetic tree and table of genetic distance [p-values] of the nucleic acid sequences of Trypanosome species in this study area and those from the region [East Africa or Africa].
ii. . I would suggest that you format tables 2 and 3 as follows;-
a) A table showing the prevalence of different trypanosome species in the study area using different diagnostic techniques [Table 2]
b) Predictors of prevalence with different trypanosome species [Table 3]. Here I would recommend the prevalence estimates to be by PCR which was the best performing diagnostic test. These are the estimates you would map [spatial distribution of different trypanosome species in the wildlife-livestock interface of Akagera region, Rwanda; see comment 1 (v) above.
c) Please delete current table 6 as the contents of this table will now be written out as one or two lines within the table 2 legend

I have provided exemplars of Table 1-2 that I would request you feel free to adopt and improve

Table 1 | Prevalence of different Trypanosome species by different diagnostic tests and agreement levels between the tests

| Trypanosome species detected | Diagnostic test [n= 1,037] | | | | | | | | | Level of agreement [Cohen Kappa value] | | |
| --- | --- | --- | --- | --- | --- | --- | --- | --- | --- | --- | --- | --- |
|  | Thin blood smears | | | Buffy coat smears | | | HRM-PCR | | |  |  |  |
|  | Crude prev | 95% CI | Adjusted [True] Prev* | Crude prev | 95% CI | Adjusted [True] Prev* | Crude prevalence | 95% CI | Adjusted [True] Prev* | Smear vs buffy coat | Smear vs HRM-PCR | Buffy coat vs PCR |
| Any Trypanosome spp |  |  |  |  |  |  |  |  |  |  |  |  |
| *T. congolense* |  |  |  |  |  |  |  |  |  |  |  |  |
| *T. vivax* |  |  |  |  |  |  |  |  |  |  |  |  |
| *T.b. brucei* |  |  |  |  |  |  |  |  |  |  |  |  |
| *T. evansi* |  |  |  |  |  |  |  |  |  |  |  |  |
| *T. Theileri* |  |  |  |  |  |  |  |  |  |  |  |  |

*Adjusted for differences in sensitivity and specificity of different tests to detect different trypanosome species [Include these here and references]

Include the comparison of sensitivity and specificity of different diagnostic tests to detect different trypanosome species in this study within this table legend [Just keep this very snappy]

**Table 2 | Predictors of infection with different trypanosome species [Positive by any test] in the wildlife-livestock interface of Akagera region, Rwanda**

| Predictor | Sampled  [n] | Any Tryp: #[%] | Tc: #[%] | Tv  #[%] | Tbb  #[%] | Te  #[%] | Tth  #[%] | Crude estimates  Based on % Any Tryp | | Adjusted estimates^γ^ | | |
| --- | --- | --- | --- | --- | --- | --- | --- | --- | --- | --- | --- | --- |
| **1.Sector herd** |  |  |  |  |  |  |  | PRR | P value* | PRR | P value* | 95% CI |
| Ndego | 70 |  |  |  |  |  |  |  |  |  |  |  |
| Kabale | 6 |  |  |  |  |  |  |  |  |  |  |  |
| Rwinkavu | 10 |  |  |  |  |  |  |  |  |  |  |  |
| Mwiri | 80 |  |  |  |  |  |  |  |  |  |  |  |
| Gahini | 89 |  |  |  |  |  |  |  |  |  |  |  |
| Murundi | 120 |  |  |  |  |  |  |  |  |  |  |  |
| Rwibongo | 190 |  |  |  |  |  |  |  |  |  |  |  |
| Kabarole | 46 |  |  |  |  |  |  |  |  |  |  |  |
| Karangazi | 174 |  |  |  |  |  |  |  |  |  |  |  |
| Rwimiyaga | 186 |  |  |  |  |  |  |  |  |  |  |  |
| Matimba | 24 |  |  |  |  |  |  |  |  |  |  |  |
| Cattle sex |  |  |  |  |  |  |  |  |  |  |  |  |
| Female |  |  |  |  |  |  |  |  |  |  |  |  |
| Male /Neutered |  |  |  |  |  |  |  |  |  |  |  |  |
| **2. Cattle breed** |  |  |  |  |  |  |  |  |  |  |  |  |
| Ankole [Local] |  |  |  |  |  |  |  |  |  |  |  |  |
| Friesian [Exotic] |  |  |  |  |  |  |  |  |  |  |  |  |
| Sanga x Friesian |  |  |  |  |  |  |  |  |  |  |  |  |
| **3. Age** |  |  |  |  |  |  |  |  |  |  |  |  |
| 6–12 months |  |  |  |  |  |  |  |  |  |  |  |  |
| 13-24 months |  |  |  |  |  |  |  |  |  |  |  |  |
| 25-36 months |  |  |  |  |  |  |  |  |  |  |  |  |
| >36 months |  |  |  |  |  |  |  |  |  |  |  |  |

^*^ Statistically significant predictor of infection with *Any trypanosome species*

**^γ^** Adjusted for predictors associated with p <0.2 at bivariate analysis

PRR= Prevalence rate ratio. You can also use OR but there an indication in literature that RR is better than OR < https://doi.org/10.3389/fvets.2017.00193 >

Xx: Change to the breeds of cattle that were included in the trial

3. **Discussion section**
i. Please revise this section to align it to the improved results section.
ii. I don’t find any discussion about the use of the panel of the diagnostic techniques used, predictors of infection [age, sex, breed, site (management system at each site)] with different trypanosomes, relative abundance of different trypanosomes [e.g., Why is *T. congolense* the most abundant and T.b.b least abundant? What kind of ecoepidemiology favours this balance? What does this ecoepidemiology indicate about mechanical transmission of AAT in this area?
iii. Unless it is the prescribed journal format, please do away with the subtitles in the discussion section. Provide contents of each of your discussion points as a paragraph
B. **Minor changes**
i. Check that the financial disclosure in the manuscript submission portal is similar to that in lines 562-602
ii. Line 19; ….” threaten both” …what do they threaten?
iii. Line 21….” the endemic area”,…..should be AAT endemic area
iv. Line 23,…”their diversity”,….Could this be genetic diversity?
v. Line 29….” parametric tests” …...just mention these tests here
vi. Line 32……..”prevalence”….prevalence of what?
vii. Line 40….. diversity”,. Change this to species
viii. Lines 43-44: improve this sentence. Why recommend control efforts to target Akagera region yet you did not screen cattle from other livestock production agro-ecological regions of Rwanda? You can argue that because this is a known tsetse belt but mechanically transmitted trypanosome species e.g., *T. vivax* often occur outside tsetse belts
ix. Line 56: Please qualify the word impact in this sentence
x. Line 61; diversity should be changed to genetic diversity
xi. Lines 68-70; I can’t seem to figure out how these datasets will help in validation of rHAT elimination given that only cattle were sampled. How about shoats, pigs, tsetse vectors? Please qualify this statement
xii. Line 75; Please just name these animal-infective trypanosomes referred to here
xiii. Line 78. You already chose to use AAT for African Animal trypanosomiasis; please use this abbreviation consistently
xiv. Please improve the sentence in lines 84-88 and make sure that all sentences in this MS follow SVO English rule. Some of the sentences in this MS are long and hard to understand. Check also sentences in lines 97-100, 121-124,446-453
xv. Lines 122-231; characterising should be changed to identifying
xvi. Lines 134-137: describe the cattle breeds studied
xvii. Lines 157-158. Explain why calves below 6 months of age are less likely to be infected with trypanosomes. Other studies < <https://doi.org/10.1371/journal.pntd.0003284>> in the region have reported high prevalence [12%] of AAT in this age group
xviii. Lines 166-167; please mention the cattle breeds. Exotic or indigenous does not mean much to readers that are not familiar with livestock breeds in Rwanda
xix. Lines 190-191; How does extracting genomic DNA cut costs? Why were two batches of DNA from one sample necessary?
xx. Line 200. Please check the holding time for enzyme activation and confirm that it was 12 minutes. Seems quite lengthy for me
xxi. Lines 255-257: Include gene accession numbers for sequences generated from this study
xxii. Lines 275-276: over all prevalence of what?
xxiii. Line 297-298 Change cases to infections
xxiv. Line 424: it could either be the microscopy or the microscopist!
xxv. Lines 454-457. Please mention what these breeds were
xxvi. Lines 494-497; sentence in here does not make sense given that apparently healthy animals were sampled. do you have the clinical picture of the sampled animals that indicated that a fraction of these animals were probable AAT cases?
xxvii. Lines 532-555. Please summarise this conclusion. Lines 557-560 should be deleted because this part of the conclusion is not supported by the study design
xxviii. Text In lines 564-566 and that in 576-580 is repeated. Delete one of these texts.
xxix. Lines 587-589. This is supposed to be in materials and methods. Section on ethics approval but not in acknowledgements
