# Supplementary material for: Occurrence, diversity and distribution of Trypanosoma infections in cattle around the Akagera National Park, Rwanda
Source: PLoS Negl Trop Dis. 2021 Dec 15;15(12):e0009929. doi: 10.1371/journal.pntd.0009929 (PMC8726506; doi:10.1371/journal.pntd.0009929)
Supplement: Attachment — Submitted filename: Rebuttal_PLoS NTDs_Gashururu.docx [file pntd.0009929.s003.docx]

Dear editor,

PLOS Neglected Tropical Diseases

**Re: Addressing the reviewers’ comments on our manuscript (PNTD-D-21-00831) - [EMID: 7ac5f11d28feee31], entitled "Occurrence, diversity and distribution of Trypanosoma infections in cattle around the Akagera National Park, Rwanda"**

We are thankful for the detailed review; we would like to submit a revised version of the manuscript in which we made the changes requested by the reviewers. We also would like to provide detailed answers to all questions and concerns.

Below are the reviewers’ comments together with their respective responses.

| **Reviewer #1**:  We are thankful for a very thorough job at reviewing our manuscript, we are therefore grateful for his input and effort aimed at improving the quality of our paper.  **A. Major changes [non-discretionary]** | | | |
| --- | --- | --- | --- |
| **Section** | **SN** | **Comment** | **Response** |
| 1. **Design/**  **methods** | i. | Gashururu et al need to include a section of sample size determination. I hope the readers should be to know some of the key parameters that were considered to calculate the sample size n=1,037.  These parameters might include but are not limited to;-  a)Number of cattle in the area  b)sectors within districts and number of cattle containing sectors in the study area  c)total number of farming households within the study area  d)Type of sampling [e.g., simple random sampling or cluster sampling]  e) If cluster sampling was adopted, what was the total number of clusters in the study area and what is the definition of the cluster? What was the minimum number of clusters that was picked to satisfy the set precision? How many animals were picked from each cluster?  f) Average number of cattle per cluster  g)sampling units/clusters [e.g., herds, or district sectors or even individual animals] and sampling fraction  h) if cluster sampling was adopted what was the intercluster correlation coefficient used?  i)background prevalence of AAT in the area if known or was a 50% baseline prevalence assumed?  j) precision of the sample estimate (one half-length of the 95% confidence interval)  k)design effect  l) Selection of each sampled animal; making sure that each animal was given an equal opportunity to be included in the survey, if indeed animals were selected randomly.  m)other [predictors of infection] data that was collected accompanying the blood samples e.g., age, sex, breed, predisposing factors [e.g., use of pyrethroid insecticides and trypanocides]  n) numbers and categories of other livestock that could be reservoirs of human infective trypanosomes or whose production/health might be compromised by AAT  o) define what indigenous, exotic, and cross breeds are and the sampling fraction for each of these breeds [i.e., total in the study area vs those that were sampled]. A good description of the inclusion criterion. Refer to lines 157-158; Please provide justification /reference for this. | This comment was addressed under the subsection ‘’Study design and sample size determination’’ of the methods. However, not all the parameters suggested by the reviewer were included. We only considered those that were applicable for our study.  The following paragraph was added:  A cross sectional study was undertaken between March and July 2019, using a stratified multistage random sampling method. The sample size was calculated using the formula for indefinite population according to Thrusfield (34). It was determined based on the baseline-estimated prevalence of 50%, with an absolute desired precision of 5% at the confidence interval of 95%. There was no reliable data on farm records, farming households and cattle population. To increase the chance of sampling many animals in whole study area, the same calculations were made for each of the three main target districts (stage 1): Kayonza (n=384), Nyagatare (n=384) and Gatsibo (n=236). The target sample size in Gatsibo District was not reached because we could not get the consent from some farmers.  We purposively selected sectors (stage 2) based on their proximity to Akagera Park, its connections and adjacent game reserves in Tanzania. In that way, one sector of the fourth District (Kirehe) was later included (n=33), thus making a total sample size of 1037 cattle. These comprised 521 (50.24%) Ankole, 514 (49.56%) Ankole x Friesian and 2 (0.19%) pure Friesian cattle. There were more females (n=946) than males (n=91) and the majority were above 2 years of age (n=876). In total,12 sectors were included in the study (6 in Kayonza, 2 in Gatsibo, 3 in Nyagatare and 1 in Kirehe Districts), and therefore considered as the strata. |
|  | i. | Motivate the readers in the background and methods sections why it was necessary to use microscopy [Thin & Buffy coat smears] and VerYDiag to detect trypanosomes or current/previous exposure to Trypanosomes. What is the value of comparing HRM-PCR, Microscopy [Thin & Buffy coat smears] and VerYDiag? Is it not obvious that HRM-PCR is better than microscopy and VerYDiag in detecting different trypanosomes? The deployment of the selected panel of diagnostic techniques is not discussed either. | A motivational paragraph was added in the background. We did not repeat the same information in methodology to avoid the duplication and keep the logical flow. Each technique was separately discussed in details under the discussion.  The following statement was added:    ‘’A number of diagnostic tests are used to detect trypanosomes, i.e. parasitological (32), Immunological (33), (34) and molecular techniques (35). Although different diagnostic tests for trypanosomes differ in sensitivity, each technique presents its own advantages and drawbacks (36), (33), (37); (38). However, their respective results may serve diversely according to the purpose. The parasitological methods are already in use and the immunological VerY Diag is commercialised in the area. PCR has not been systematically used for the detection of trypanosomes in Rwanda. Nevertheless, respective test results would purposively inform farmers and other relevant stakeholders according their needs.’’ |
|  | ii. | Given the multiplicity of the diagnostic/ analytical procedures undertaken, it would be beneficial to include a schematic study flow diagram so that readers can quickly visualise what was done to arrive at the results presented herein. | Addressed – the schematic study flow was included towards the end of the methodology section. |
|  | iii. | The authors need to consider undertaking phylogenetic analysis [Gamma distribution with 1000 bootstrap replicates] of the nucleic acid sequences of Trypanosome species in this study area and those from the region [East Africa or Africa]. In addition, it would be nice to evaluate sequence divergence [pairwise genetic distances] of Trypanosome species in this study and their corresponding reference sequences | The phylogenetic tree and the evaluation of trypanosome species divergence were added as suggested. |
|  | iv. | I would like to suggest that Figure 3 is revisited with a view to map the spatial distribution of T. vivax, T. congolense, Trypanozoon species and T. theileri using either of the following strategies  a) Aggregated farm level prevalence of different Trypanosome species detected by the most sensitive /specific diagnostic test or an average of all tests; with the diameter of the circle showing prevalence proportional to sampled animals or prevalence symbology stratified like 0-5, 5-10, 10-15, 15-20; with increasing size of the symbology. If the number of sampled farms is too many, you can aggregate these data sets at sector level  b) You can map presence and absence of different species, where presence and absence are as determined by all diagnostic tests or the most sensitive/specific test  Note that option (a) is more intuitive than option (b) | This is a good advice; however, the study was not designed for this. We tried mapping the prevalence and clustering was not used. There is a trade-off between presenting aggregated/clustered prevalence data, and mapping disaggregated absence/presence data. For this study, we preferred the latter, because (1) we gave priority to the accuracy of the spatial distribution, and (2) prevalence data are provided in tables.  However, we have added the prevalence and the symbology as suggested. The figure is now number 4, not 3. |
| **2. Results** | i. | Please include results related to comment 1 (iv) above, i.e., phylogenetic tree and table of genetic distance [p-values] of the nucleic acid sequences of Trypanosome species in this study area and those from the region [East Africa or Africa]. | The phylogenetic tree and the evaluation of trypanosome species divergence were added as suggested. |
|  | ii. | I would suggest that you format tables 2 and 3 as follows;-  a) A table showing the prevalence of different trypanosome species in the study area using different diagnostic techniques [Table 2]  b) Predictors of prevalence with different trypanosome species [Table 3]. Here I would recommend the prevalence estimates to be by PCR which was the best performing diagnostic test. These are the estimates you would map [spatial distribution of different trypanosome species in the wildlife-livestock interface of Akagera region, Rwanda; see comment 1 (v) above.  c) Please delete current table 6 as the contents of this table will now be written out as one or two lines within the table 2 legend  I have provided exemplars of Table 1-2 that I would request you feel free to adopt and improve | We are thankful for the reviewer’s input concerning the improvement of the tables. We formatted the tables as suggested, however, we later realized that the aim of our study was diverted and may consequently affect the entire buildup of the paper. It may therefore change the message we wish to convey.  Following are some reasons:  1. The proposed format does not allow discrimination of pathogenic trypanosomes and non-pathogenic ones. This heavily affects the prevalence rates of AAT in different sectors. Some sectors had almost only non-pathogenic species in high numbers and their prevalence became alarming inappropriately. Others are really affected by different pathogenic trypanosomes but it cannot be seen because of the moderate prevalence. Veterinary services and policy makers are interested in pathogenic species. This information may mislead them during control activities.  2. The proposed format does not take into account the mixed infections at all. They are nowhere shown.  3. The microscopy is not able to differentiate Trypanozoon species (*T.brucei* and *T.evansi* in our case). Those 2 species were only resolved by PCR. In the proposed tables, those species are separate and we are not able to provide their values (prevalence and statistical data). The same applies to *T. theileri*, which was not yet identified at microscopy.  4. All the maps presented in the manuscript were designed according to our objectives to show the distribution / occurrence of pathogenic and non-pathogenic species separately. We may have to revisit them to match the new format.  5. The new tables do not consider showing the sensitivity and specificity of the VerY Diag technique in comparison to the techniques used. VerY Diag is being used in the area and we would like to show comparative results to their users.  6. This is a baseline study, and the first of its kind in the area. The findings are meant to inform further related studies. It is not generally easy to apply models in a baseline study where some important information is still missing. We would wish to apply the relative risk assessment between different predictors in our future studies, where each relevant information (clusters and their representative population, etc.) will be taken into account.  However, we revisited the initial table 3 to include other predictors proposed by the reviewer (sex, breed, age). |
| **3.Discussion** | i. | Please revise this section to align it to the improved results section. | This was affected by the above explanations |
|  | ii. | I don’t find any discussion about the use of the panel of the diagnostic techniques used, predictors of infection [age, sex, breed, site (management system at each site)] with different trypanosomes, relative abundance of different trypanosomes [e.g., Why is T. congolense the most abundant and T.b.b least abundant? What kind of ecoepidemiology favours this balance? What does this ecoepidemiology indicate about mechanical transmission of AAT in this area? | The discussion section was extensively revised to include all the reveiwers’comments, including these ones.  The current logical flow of the discussion goes as follow: species / abundance, mechanical transmission, diagnostic techniques used, relevant predictors, PCV and SRA gene absence. |
|  | iii. | Unless it is the prescribed journal format, please do away with the subtitles in the discussion section. Provide contents of each of your discussion points as a paragraph | Addressed, subtitles were removed from the discussion. |
| **B. Minor changes** | | | |
|  | i. | Check that the financial disclosure in the manuscript submission portal is similar to that in lines 562-602 | It is well noted, we will harmonise this during the resubmission process. |
|  | ii. | Line 19; ….” threaten both” …what do they threaten? | Addressed ‘’ threaten the life of both humans and animals |
|  | iii. | Line 21….” the endemic area”,…..should be AAT endemic area | Addressed : AAT was added |
|  | iv. | Line 23,… ”their diversity”,….Could this be genetic diversity? | Addressed : genetic diversity |
|  | v. | Line 29….” parametric tests” …...just mention these tests here | ANOVA was added |
|  | vi. | Line 32……..”prevalence”….prevalence of what? | Addressed: ‘’prevalence of trypanosome infections’’ |
|  | vii. | Line 40….. diversity”,. Change this to species | Addressed : diversity was changed to species |
|  | viii. | Lines 43-44: improve this sentence. Why recommend control efforts to target Akagera region yet you did not screen cattle from other livestock production agro-ecological regions of Rwanda? You can argue that because this is a known tsetse belt but mechanically transmitted trypanosome species e.g., T. vivax often occur outside tsetse belts | The sentence was removed as it was not supported by the study findings.  The remained statement is ‘’The study confirms that the area around the Akagera NP is affected by AAT, and should therefore, be targeted during the control activities.’’ |
|  | ix. | Line 56: Please qualify the word impact in this sentence | Addressed: ‘’socio-economic and health impact …’’ |
|  | x. | Line 61; diversity should be changed to genetic diversity | Addressed as suggested |
|  | xi. | Lines 68-70; I can’t seem to figure out how these datasets will help in validation of rHAT elimination given that only cattle were sampled. How about shoats, pigs, tsetse vectors? Please qualify this statement | We thank the reviewer for the concern raised. However, in the ‘Author’s abstract’, words are limited and we have too few words to explain this in detail. Therefore, It is not possible to address the suggestion in the manuscript.  That said, the statement means that:   (1) Data on the presence (or absence of detection) of *T. b. rhodesiense* in non-human animals gives insights into the general circulation of the parasite. (2) The reviewer is right that data on *T.b.rhodesiense* absence of detection do not, by themselves demonstrate the elimination of rHAT, but they do provide very useful complementary information. (3) As demonstrated by already validated dossiers of HAT elimination as a public health problem at the national level (e.g. Togo, Côte d’Ivoire), HAT surveillance data in humans is crucial to demonstrate elimination as a PHP. However, data in non-human animals provide useful complementary data, which is particularly useful in rHAT regions (East and South Africa). It is true that also data from ‘goats, pigs, tsetse vectors’ would also be useful, but this does not mean that data from cattle are not useful. |
|  | xii. | Line 75; Please just name these animal-infective trypanosomes referred to here | Addressed: ‘’*T.vivax* in particular’’ was added. |
|  | xiii. | Line 78. You already chose to use AAT for African Animal trypanosomiasis; please use this abbreviation consistently | Addressed: ‘’AAT’’ was used instead of Glossina-transmitted Trypanosomiasis |
|  | xiv. | Please improve the sentence in lines 84-88 and make sure that all sentences in this MS follow SVO English rule. Some of the sentences in this MS are long and hard to understand. Check also sentences in lines 97-100, 121-124,446-453 | The sentences were revised as suggested by reviewer. The long ones were reformulated to facilitate the understanding. |
|  | xv. | Lines 122-231; characterising should be changed to identifying | Addressed: Characterising was changed to ‘’identifying’’. Unless it is typing error to mean 123, the word was not found at line 231. |
|  | xvi. | Lines 134-137: describe the cattle breeds studied | Addressed : Indigenous= Ankole, crossbreeds= Ankole x Friesian and Exotic= Friesian |
|  | xvii. | Lines 157-158. Explain why calves below 6 months of age are less likely to be infected with trypanosomes. Other studies < https://doi.org/10.1371/journal.pntd.0003284> in the region have reported high prevalence [12%] of AAT in this age group | The following statement was added: ‘’…, considering the adopted local management system. Farmers do not take their young calves to risky areas for grazing. |
|  | xviii. | Lines 166-167; please mention the cattle breeds. Exotic or indigenous does not mean much to readers that are not familiar with livestock breeds in Rwanda | Addressed, the term ’’breeds’’ was clarified across the whole document. Indigenous= Ankole, crossbreeds= Ankole x Friesian and Exotic= Friesian |
|  | xix. | Lines 190-191; How does extracting genomic DNA cut costs? Why were two batches of DNA from one sample necessary? | We thank the reviewer for seeking clarification about this.  The statement might have sound differently. Due to logistic challenges during early COVID-19 time, we could not get enough Bioline kits to finish all the 1037. The cost of getting a kit was not affordable, reason why we decided to use another extraction method. We used the bioline kits for around the half of the samples, and the non-enzymatic method for the remaining samples. It is not 2 different extraction methods for the same sample. All the methods used give a quality DNA for further molecular steps.  The extraction method section was revisited to clarify how we used two extraction methods. |
|  | xx. | Line 200. Please check the holding time for enzyme activation and confirm that it was 12 minutes. Seems quite lengthy for me | 12 minutes time is ideal. The enzyme used is EVAGREEN which a hot start that requires an initial activation at a higher temperature of 95 degrees between 12 – 15 minute, according to the manufacturers’ instructions. |
|  | xxi. | Lines 255-257: Include gene accession numbers for sequences generated from this study | The generated accession numbers were included in the results under the section ‘’ Genetic diversity of trypanosome species’’. |
|  | xxii. | Lines 275-276: over all prevalence of what? | Addressed : ‘’…of trypanosome infections’’ |
|  | xxiii. | Line 297-298 Change cases to infections | Addressed as suggested |
|  | xxiv. | Line 424: it could be either the microscopy or the microscopist! | Addressed: the microscopy was preferred. |
|  | xxv. | Lines 454-457. Please mention what these breeds were | Addressed, the term ’’breeds’’ was clarified across the whole document |
|  | xxvi. | Lines 494-497; sentence in here does not make sense given that apparently healthy animals were sampled. do you have the clinical picture of the sampled animals that indicated that a fraction of these animals were probable AAT cases? | Comment addressed: the sentence was removed. |
|  | xxvii. | Lines 532-555. Please summarise this conclusion. Lines 557-560 should be deleted because this part of the conclusion is not supported by the study design | The conclusion was revised. Some statements were removed. The statement that was not supported was reshuffled to meet the study design |
|  | xxviii. | In lines 564-566 and that in 576-580 is repeated. Delete one of these texts. | The repeated sentence was deleted |
|  | xxix. | Lines 587-589. This is supposed to be in materials and methods. Section on ethics approval but not in acknowledgements | The statement on ethical clearance was shifted to the methodology section as suggested. |
| **Reviewer #2** | | | |
| **Conclusion** |  | Some limitations are mentioned here and there, but there is not a paragraph focusing on limitations, which could be helpful as guidance for further studies | A paragraph on study limitations was added at the end of the discussion section. |
| **Abstract** |  | About the PCV, authors probably mean to say no significant difference instead of no statistical significance.  The statement “should target mainly areas around Akagera NP” is not a conclusion supported by the findings, because this study only surveyed those areas.  Similarly, the statement “to advance in the Progressive Control Pathway (PCP)” is not a conclusion of this study that doesn’t deal with control strategies at all. | Addressed: ‘’There was no significant difference statistical significance between the mean PCV of infected and non-infected animals.’’  The statement was amended and the following sentence was added: ‘’The study confirms that the area around the Akagera NP is affected by AAT, and should therefore, be targeted during the control activities.’’  “We, authors believe that this statement is pertinent in the context of the study conclusions.  In a scientific paper, conclusions do not have to focus only on the scientific aspects of the study, but they can, and indeed should, address the broader relevance of the study. In addition, the PCP should not be regarded too narrowly as a ‘control strategy’. It is also, and indeed most crucially, an approach to planning, where the importance of epidemiological information for evidence-based decision-making is strongly promoted. In this light, our study provides a good example of baseline information that can be collected to inform decision making for the progressive control of AAT. |
| **Methods** |  | Explain why PCV was measured (e.g. to check correlations of anaemia with trypanosomes?). | The following sentence was added: ‘’The PCV was measured to check correlations of anaemia with trypanosome infections.’’ |
| **Discussion** |  | There were less infections in crossbreeds than indigenous cattle. The authors point to a likely explanation (farmers care more about the improved cattle than indigenous ones) which makes good sense, but can they add if there are data showing different susceptibility to trypanosome infection between these types of cattle? If yes, how does it align with these findings?  It looks contradictory that after authors describe T. theileri as non-pathogenic and benign, they recommend investigations to evaluate the effect of T. theileri on cattle and /or other livestock.  When talking about surveillance, it seems correct to say that it may confirm the absence of rhodesiense HAT, but it is less clear how it can predict the potential re-emergence of the disease in the area. Perhaps authors mean to say that it can detect a re-emergence.  The statement “Looking at the infection distribution, Akagera NP seems to be the main source of infection” may come from other data, but it doesn’t appear to derive from this study, because the only area investigated was around Akagera NP, and the authors don’t analyse or show a difference of distance to the park of the sites with trypanosomes versus without. The maps don’t show that clearly either. And actually the contrary seems true for Tbb, found closer to Ibanda reserve than to Akagera NP. | The following statement was added: ‘’However, there is no previous data showing different susceptibility to trypanosome infection between these types of cattle breeds in the area to align with our findings. ‘’  It is believed that *T.theileri* is non-pathogenic to cattle. Our recommendation is to re-evaluate the pathogenic effect of T.theileri group to cattle and other livestock in the area. Changes may occur with the time. The statement was fine-tuned to make the recommendation clear: ‘’ Further investigation on this is recommended to evaluate the current pathogenic effect of *T. theileri* group effect on cattle and /or other livestock species health.’’  The verb ‘’predict’’ was changed into ‘’detect’’ to give the right sense of the statement.  The statement was reshuffled to meet the study findings as follow: ‘’ Looking at the infection distribution, areas around Akagera NP as well as areas bordering the game reserves in Tanzania should be targeted for control and prevention strategies. ‘’  In this study, the focus was only around the park. The distance to the park could not really reflect the exposure because animals move often from one place to another. That is the reason why we did not analyse the effect of the distance. |
| **Reviewer #3** | | | |
| **Conclusion** |  | The conclusions are supported by the data. The limitations of the study could be slightly more elaborated (such as lack of data for the rest of Rwanda). | A paragraph on study limitations was added at the end of the discussion section. |
| **Abstract** |  | Line 20: the sentence “The diseases occur in poor and vulnerable settings of Africa.” Is very general and can be removed. In the results section, a sentence about occurrence of trypanosomes in indigenous versus crossbreed animals might be added as this is a parameter of interest which was also studied. | Sentence “The diseases occur in poor and vulnerable settings of Africa” was removed from the abstract.  The following sentence was added in the abstract: ‘’Lower trypanosome infections were observed in Ankole x Friesian breeds than indigenous Ankole,’’ |
| **Introduction** |  | Line 107-109: The sentence: “Because of this epidemiological situation and the existence of an adequate surveillance system, Rwanda as a country meets the requirements to apply for WHO validation of HAT elimination as a public health problem at the national level (13). Currently, the application process for Rwanda is in progress.” Comes a bit early in the introduction and would be more appropriate in the discussion in the section about Trypanosoma brucei rhodesiense. Mention of an adequate surveillance system can be done in the previous sentence. | Thank you for the advice, the following statement was shifted from introduction to the discussion under the paragraph about *T.b.rhodesiense*.  ‘’Rwanda as a country meets the requirements to apply for WHO validation of HAT elimination as a public health problem at the national level (13). Currently, the application process for Rwanda is in progress.’’  ‘’…adequate surveillance ...’’was incorporated in the previous sentence as suggested. |
| **Methods** |  | Line 135: mention the names of the indigenous and cross breed cattle | This was addressed across the document. Indigenous = Ankole, crossbreed= Ankole x Friesian |
|  |  | Line 145: 145 Remove the term “warmer” in the sentence as this should be relative to something elso. The temperature range is already given a few sentences later. | The term ‘’warmer’’ was removed from the sentence. |
|  |  | Line 172: when was BCT carried out and when were specimens for PCR prepared | The following sentence was added:  ‘’At the same laboratory, the buffy coat technique (BCT) was carried out and the specimen for PCR were prepared.’’ |
|  |  | Line 179: how were smears stained and what was microscopic magnification used to detect trypanosomes? | The staining and magnification were added in the same sentence. |
|  |  | Line 195: include a reference to table 1 in the section | The reference to table 1 was included in the section. |
|  |  | Line 246: in the table add a column indicating for each primer species/subspecies specificity | The primer specificity was added in the column 2 along with the target gene. |
|  |  | Line 258: was VerY Diag carried out on fresh blood specimens or stored ones? Give a detailed protocol, including the volume of the blood added. | The following statements were included in the paragraph:  ‘’The test was done on fresh blood samples after collection.’’  And  ‘’With the help of a pipette supplied in the kit, a drop of whole blood (20 μL) was deposited into the specimen well of the cassette. Immediately, another drop (40 μL) of the dilution buffer was added to the same well, avoiding to drop any solution in the cassette observation window. Results were read after 10 minutes.’’ |
| **Results** |  | Line 301: in the table it is difficult to see which column corresponds to which technique | To address this, the table design was changed to allow clear columns separation. Additionally, we assume the graphic designers will revisit the table formatting when the manuscript is accepted and the PDF prepared, according to the journal requirements. |
|  |  | Line 308-314: Figure 2 & 3 summarize very well the result. However the color choice for the Trypanozoon panel is not optimal, as the green spots are hardly visible. Choice of another color is indicated. | Comment well received. We have revised the figures and made the grey point lighter, thus making the green points more visible. |
|  |  | Line 315: add significance levels when comparing breeds | The P value was added (p=0.000).’ |
|  |  | Line 333 figure 5: font size of the text of the graphs should be increased. | We have a challenge of images generated by QuantStudio 3 not being editable. We grouped them together in one image to show different melt curves according to the species. We would like to keep the same figure for the sake of our presenting purpose. |
|  |  | Line 351: How many animals in the non infected group had a PCV below the 26% threshold value? Add a line “non infected” in table 4. | The following statement was added:  ‘’In non-infected group, 209 animals had the PCV below the threshold while 551 animals were above 26%’’. |
|  |  | Is there anything to mention about occurrence of trypanosomes or presence of antibodies in function of animal age? | The following sentence was added under VerY Diag results: ‘’Trypanosome antibodies were detected more in adult cattle (168/296) than young ones (16/296).’’ |
| **Discussion** |  | Comment on trypanosome “hotspot” in Karangazi (the north West tip of Agagera?), and in particular on T evansi.  How does T evansi presence in Rwanda compare to surrounding countries? Would there be an interest to screen for T evansi antibodies for example using CATT/T evansi in Rwanda (also in the light of presence of T evansi subtype A only)? | The hotspot in Karangazi was earlier discussed under this statement:  A cluster of Trypanozoon species was noted in the north of Akagera NP (Fig 3). The north of the park has a higher concentration of wild animals (19) (20) and the same is the case for livestock. Similarly, there are higher densities of tsetse flies (16) and other biting flies, hence an increased transmission. There might be some preferred hosts harbouring Trypanozoon species on which the flies and other biting flies are feeding.  Additionally, we revised the statement on *T. evansi* as follows (first paragraph of discussion):  *T. evansi* has a wide host range (33) and similar sub-type A was isolated from camels and buffalo in the region (48)). Regionally, this trypanosome species was extensively found and studied in Kenya (48),(49). Infections of *T. evansi* might be mistaken with other trypanozoon species. We recommend a specific, cheaper field serological test CATT/T.evansi in the area, because PCR is expensive and therefore, not affordable locally. |

ADDITIONAL COMMENT FROM AUTHORS

- Due to revisions, line numbers changed. The revised version is longer than the initial submitted manuscript. Revisions are seen in track changes. The discussion was grossly reshuffled, but all the reviewers’comments were addressed though the line numbers changed. Paragraphs discussing the various diagnostic methods used were linked to maintain the chronology.

A short statement to align their use and its implications was added as suggested by reviewer 1.

- Line 351 of initial submission: we had mistakenly written 1941 instead of 191, it was corrected
- Line 367 of initial submission: a sentence was added for better description- The two GenBank similarities correspond to the TvL1-G genotype (West Africa & East Africa) of *T.vivax.*

Sincerely,

Richard Gashururu

The manuscript corresponding author
